# Discordance between patient and physician reported global disease activity in PsA is associated with mental health—a cross-sectional analysis

**DOI:** 10.1007/s00296-025-05933-0

**Published:** 2025-08-05

**Authors:** Anne C. Regierer, Kirsten Karberg, Cay-Benedict von der Decken, Stephanie Lembke, Carlo Veltri, Matthias Englbrecht, Johanna Callhoff, Stefan Kleinert

**Affiliations:** 1 Epidemiology and Health Services Research, German Rheumatology Research Center (DRFZ Berlin), Charitéplatz 1, 10117 Berlin, Germany; 2 RHADAR GbR, Möhrendorfer Str. 1c, 91056 Erlangen, Germany; 3 Rheumatologisches Versorgungszentrum Steglitz, Berlin, Germany; 4 Freelance Healthcare Data Scientist, Greven, Germany; 5 Praxisgemeinschaft Rheumatologie-Nephrologie, Erlangen, Germany; 6 Department of Medicine II, Rheumatology/Clinical Immunology, University Hospital of Würzburg, Würzburg, Germany; 7 Klinik für Internistische Rheumatologie, Rhein-Maas-Klinikum, Würselen, Germany; 8 Medizinisches Versorgungszentrum Stolberg, Stolberg, Germany

**Keywords:** Psoriatic arthritis, Patient reported outcome, Global disease activity, Depression, Observational data

## Abstract

Psoriatic arthritis (PsA) is a complex disease with heterogeneous disease manifestations. Composite scores including patient reported parameters are the recommended tools to measure these different aspects. Patient and physician often show discordance in their disease assessment. To describe the discordance between patient (PtGA) and physician reported global disease activity (PhGA) and to show its association with mental health. Descriptive cross-sectional analysis of three independent cohorts of PsA patients – RABBIT-SpA register, national database (NDB), RheumaDatenRheport GbR (RHADAR) network – receiving routine rheumatology care in Germany. Association between depressive symptoms and discordance between PtGA and PhGA was analysed using a linear regression model taking age, sex, and depression score into account. 1931 RABBIT-SpA, 1794 NDB, and 1728 RHADAR patients were analysed. Mean age was 55, 56, and 60 years. Mean discordance (PtGA-PhGA) was 1.8 in RABBIT-SPA, 1.9 in NDB, and 2 in RHADAR. Discordance > = 3 was present in 34% (RABBIT-SpA), 35% (NDB), and 37% (RHADAR). Discordance increased with increasing PtGA. In patients with severe depressive symptoms, discordance was larger than in those with no depressive symptoms. Depressive symptoms were significantly associated with discordance also if age and sex were accounted for. In this analysis of three independent PsA cohorts, we found a clinically meaningful discordance between physician and patient assessed disease activity in more than a third of the patients. As patients report higher levels of disease activity, discordant judgments become increasingly prevalent. Depressive symptoms are strongly associated with discordant assessments of global disease activity.

**Supplementary Information:**

The online version contains supplementary material available at 10.1007/s00296-025-05933-0.

## Introduction

Psoriatic arthritis (PsA) is a severe chronic inflammatory condition with several disease domains, often affecting skin, nails, joints, entheses, and axial skeleton, and requires timely treatment with immunosuppressive treatment after diagnosis to achieve the lowest possible level of disease activity [1].

Several instruments to measure disease activity in PsA are routinely used. Composite scores are the recommended tools to measure this heterogeneous disease and to guide treatment decisions [1]. However, there is currently no universally accepted gold standard for composite measures in PsA, leading to variability in their application [2]. The Group for Research and Assessment of Psoriasis and Psoriatic Arthritis (GRAPPA)-Outcome Measures in Rheumatology (OMERACT) initiative is actively working to standardize outcome measure for PsA, aiming to harmonize their use in randomized controlled trials (RCTs) and longitudinal studies [3]. This includes efforts to define a standardized composite measure to comprehensively assess disease activity [4].

Most composite outcome measures comprise of physicians assessments of the clinical domains, of laboratory measures (CRP or ESR), and of patient-reported outcome parameters (PRO). Therefore, PROs are used routinely to guide treatment decisions. A similar assessment of the disease activity by patients and physicians is desirable for treatment decisions. A potential discordance must be recognized and is very relevant for the management of PsA patients. Discordance has been described in PsA, with varying frequency, depending on the definition of discordance. In a large European cross-sectional study of 460 PsA patients treated in routine rheumatology care, discordance, defined as patient global disease activity (PtGA)– physician global disease activity (PhGA) > = 3 (on a 0–10 NRS), was present in almost 30% of the patients and in almost 50% using a > = 2 point cut-off [5]. In a study from the Danish national rheumatology registry (DANBIO), the PtGA was 2 or more points higher than the PhGA in 57% of the PsA patients [6]. There is consistent evidence that the mean patients’ assessment is higher than the mean physicians’ assessment [5, 7, 8].

The aim of this study is to analyze the discordance between physicians and patients assessment of disease activity in three independent cohorts of PsA patients receiving routine rheumatology care in Germany. Our hypothesis is that this discordance is associated with the presence of depressive symptoms.

## Methods

A descriptive cross-sectional analysis of three independent cohorts of PsA patients– the RABBIT-SpA register, the national database (NDB), and the RheumaDatenRheport GbR (RHADAR) network– receiving routine rheumatology care in Germany was conducted.

### RABBIT-SpA register

The German disease register RABBIT-SpA is a prospective longitudinal observational multi-centre cohort study in Germany, which started in 2017. Adult patients diagnosed by the treating rheumatologist with PsA initiating a new treatment with a biologic disease modifying antirheumatic drug (bDMARD), targeted synthetic (ts)DMARD or a conventional treatment (csDMARDs and/or NSAIDs) can be included into the register. A more detailed description of the study can be found elsewhere [9, 10]. Prior to enrolment into RABBIT-SpA, all patients gave their informed consent. RABBIT-SpA received approval by the ethics committee of the Charité-Universitätsmedizin Berlin (#EA1/246/16). For the analysis presented, data from the patients’ last visits evaluating the outcome of interest were included. Data base closure was March 1st 2024.

### National database of the arthritis centres (NDB)

The German National Database of the Arthritis centres is a long-term multi-centre observational study to describe the healthcare situation of people with inflammatory rheumatic diseases in Germany, a detailed description is published elsewhere [11]. Physician and patient-reported outcomes are collected once yearly for people with any inflammatory rheumatic disease. For this analysis data from 2022 was used. Ethics approval for the NDB was granted by the ethics committee of the Charité-Universitätsmedizin Berlin (EA1/196/06).

### RheumaDatenRhePort (RHADAR)

The German RHADAR database contains pseudonymized data collected during routine clinical care at German rheumatology centers [12]. All patients provided written informed consent before participating in the registry. Ethics approval for the study was not required by the Ethics Committee of the Bavarian State Medical Association, as it was a non-interventional quality assurance evaluation of routine medical care. For the analysis presented, data from the patients’ last visits evaluating the outcome of interest between January 5th, 2022, and August 5th, 2024, were included.

### Variables

Data were collected on sociodemographic and clinical characteristics, as well as PROs. Sociodemographic characteristics included factors such as gender, age, BMI, school education, and smoking. Clinical factors encompassed: disease duration, CRP, 66/68 swollen and tender joint count (SJC/TJC), physician global disease activity (PhGA), comorbidities. Disease Activity in Psoriatic Arthritis (DAPSA) score was used to measure disease activity.

PROs contained patient global disease activity (PtGA), impact of disease by the Psoriatic Arthritis Impact of Disease (PsAID) score as well as the individual PsAID variables, functional status via the Health Assessment Questionnaire (HAQ) or the Funktionsfragebogen Hannover (FFbH). Quality of life was assessed via the Dermatology Life Quality Index (DLQI). Depressive symptoms were assessed using the WHO-5 Well-Being Index in RABBIT-SpA and NDB. PHQ-4 was used in RHADAR. The antirheumatic treatment is grouped according to the mode of action.

### Statistical analysis

**Fig. 1:**
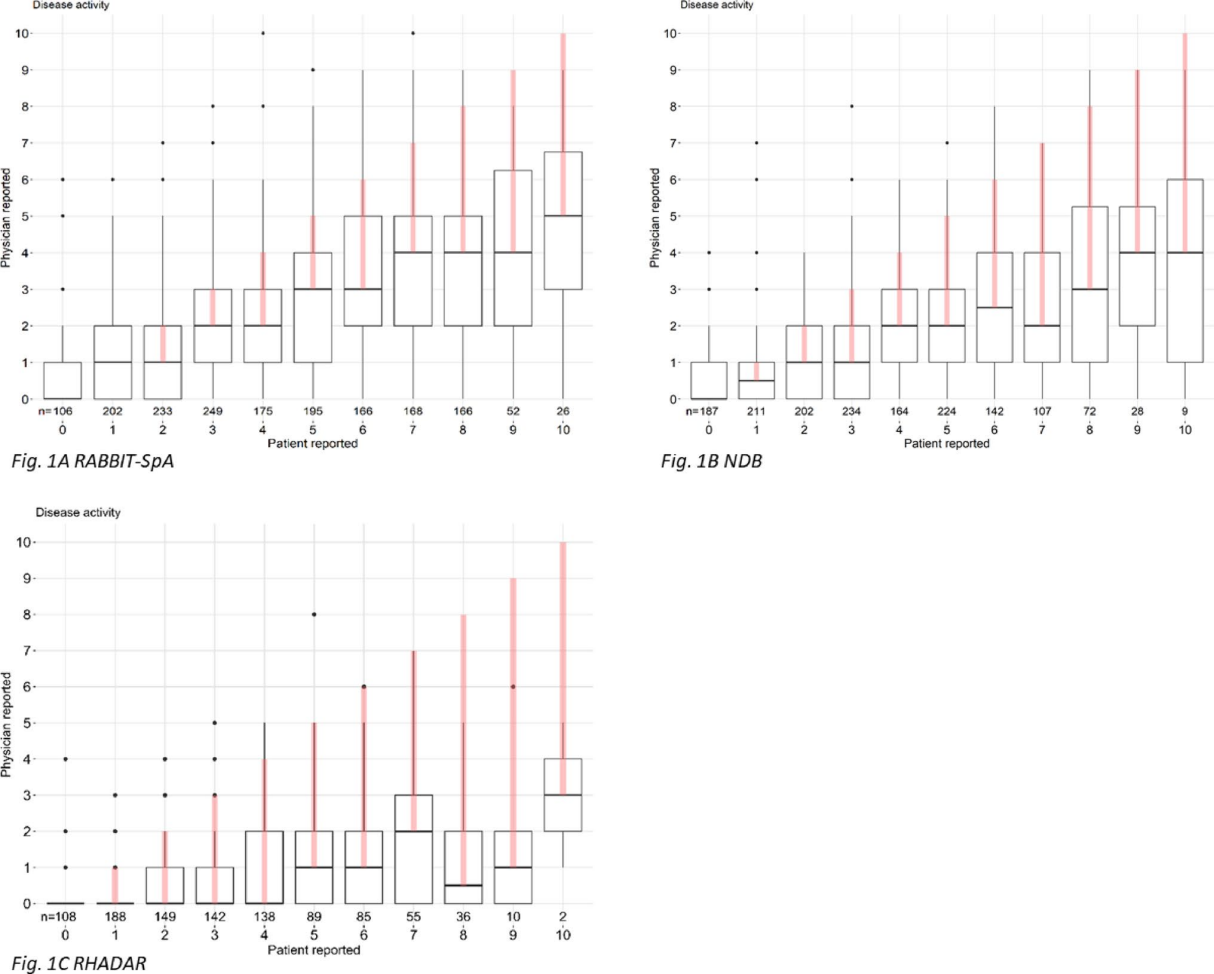
Boxplots of patient reported global disease activity (PtGA) and physician reported global disease activity (PhGA). On the x-axis PtGA values (0–10) are shown and on the y-axis PhGA values. The boxplots show the distribution of PhGA values per PtGA value. Boxes range from the 25% percentile to the 75% percentile, the line in the middle of the box shows the median. The pink line shows the discordance between the PtGA median value and the PhGA value** A** RABBIT-SpA;** B** NDB;** C** RHADAR. * NDB* national data base, * RABBIT-SpA* German disease register RABBIT-SpA, *RHADAR* RheumaDatenRhePort

**Fig. 2:**
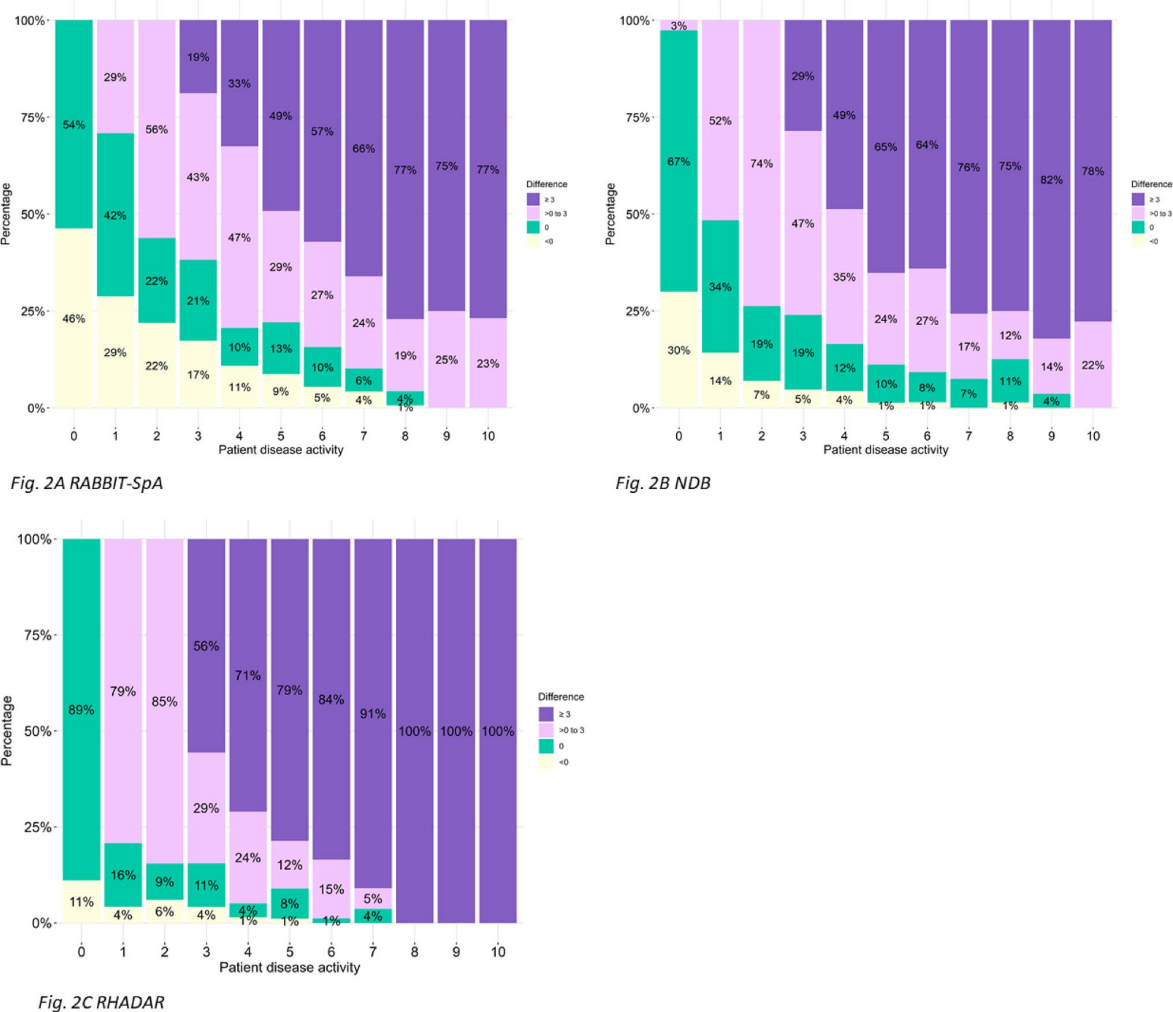
Distribution and magnitude of discordance between PtGA and PhGA. <0: PhGA higher than PtGA, > 0: PtGA is higher than PhGA. **A** RABBIT-SpA;** B** NDB;** C** RHADAR. *NDB* national data base,* RABBIT-SpA* German disease register RABBIT-SpA,* RHADAR* RheumaDatenRhePort

**Fig. 3:**
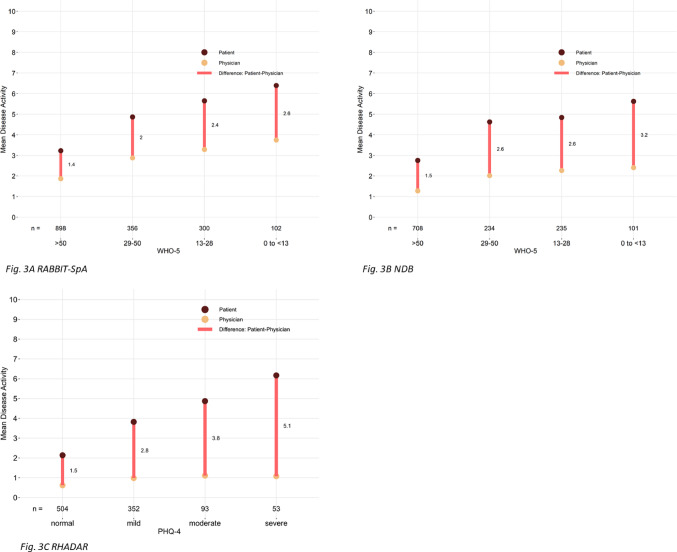
Categories of depressive symptoms and the discordance between PtGA (dark brown circles) and PhGA (light orange circles). The red lines show the mean difference between PtGA and PhGA for each category of depressive symptoms. WHO-5 > 50: well-being, 29–50: mild depressive symptoms, 13–28: moderate depressive symptoms, 0–13: severe depressive symptoms. **A** RABBIT-SpA; ** B** NDB;** C** RHADAR.* NDB* national data base,* RABBIT-SpA* German disease register RABBIT-SpA,* RHADAR* RheumaDatenRhePort,* WHO-5* WHO-5 well-being index

The characteristics of the different cohorts are shown descriptively. For each cohort, boxplots of PhGA values are shown for each PtGA value (Fig. 1). Additionally, Fig. 2 shows if PtGA and PhGA differ relevantly for each value of PtGA, with a relevant difference defined as 3 or more points difference. The difference between PtGA and PhGA values is also shown stratified by depressive symptoms (Fig. 3).

To explore if differences in PtGA and PhGA for the different levels of depressive symptoms remain after age and sex are considered, a linear regression model for each cohort was fitted. The difference between PtGA and PhGA was modelled as a linear function of age, sex and depression score (without categorization).

## Results

### Description of cohorts

The analysis was based on 1,931 RABBIT-SpA patients, 1,794 NDB patients and 1,728 RHADAR patients (Table 1). The mean age in the three cohorts was 55, 56, and 60 years; the percentage of female patients was 59%, 56%, and 55%. Disease duration was longer in the RHADAR cohort compared to RABBIT-SpA and NDB.

**Table 1:** Sociodemographic characteristics

	*n* total/*n* missing	RABBIT-SpA	*n* total/*n* missing	NDB	*n* total/*n* missing	RHADAR
*n*		1931		1794		1728
Female, n (%)	1931/0	1142 (59.1)	1794/0	981 (55.7)	1727/1	950 (55.0)
Age in years, mean (SD)	1931/0	55 (12.5)	1794/0	56.3 (13.8)	1728/0	59.8 (13.5)
BMI, mean (SD)	1810/121	29.1 (6)	1436/358	28.5 (6.0)	NA	NA
School education > = 10 years, n (%)	1709/222	1359 (79.5)	1671/123	1227 (73.4)	NA	NA
Smoking, current, n (%)	1675/256	502 (30)	1679/115	390 (23.2)	1100/628	166 (9.1)
Years since diagnosis, mean (SD)	1746/185	8.9 (8.1)	1193/576	10.6 (10.1)	1583/145	14.3 (9.9)
Number of comorbidities, mean (SD)	1924/7	2.3 (2.5)	1794/0	2.5 (1.8)	NA	NA
Number of comorbidities: >=3, n (%)	1924/7	692 (36)	1794/0	737 (41.1)	NA	NA
Comorbidity: Depression, n (%)	1924/7	262 (13.6)	1794/0	163 (9.1)	NA	NA

*BMI* body mass index, * NA* not available, * NDB* national data base, * RABBIT-SpA* german disease register RABBIT-SpA, * RHADAR* RheumaDatenRhePort, * SD* standard deviation

### Comparison of the clinical characteristics between the three cohorts

Markers of disease activity showed some differences in the three cohorts (Table 2). That is, DAPSA score, TJC, SJC, and PhGA were highest in RABBIT-SpA, followed by NDB and then by RHADAR. PROs showed a similar pattern, with worst scores in RABBIT-SpA, followed by NDB and RHADAR (Table 2).

**Table 2:** Clinical and patient-reported characteristics

	*n* total/*n* missing	RABBIT-SpA	*n* total/*n* missing	NDB	*n* total/*n* missing	RHADAR
*n*		1931		1794		1728
Tender Joint Count (0–68), mean (SD)	1908 / 23	3.2 (6.2)	1164/630	2.7 (1.6)	1680/48	1.8 (4.0)
Tender joints yes, n (%)	1908 / 23	930 (48.7)	1164/630	565 (48.5)	1680/48	675 (39.1%)
Swollen Joint Count (0–66), mean (SD)	1908 / 23	1.2 (3.3)	1164/630	0.7 (2.0)	1680/48	0.5 (1.7)
Swollen joints yes, n (%)	1908 / 23	514 (26.9)	1164/630	281 (24.1)	1680/48	302 (17.5%)
Body surface area (%), mean (SD)	1882 / 49	3.1 (7.5)	NA	NA	NA	NA
CRP mg/l, mean (SD)	1744 / 187	4.3 (11.5)	1523/271	6.2 (14.1)	1399/329	4.9 (10.2)
DAPSA, mean (SD)	1485 / 446	13.4 (11.5)	935/834	11.1 (10.1)	1248/480	8.8 (8.1)
PhGA, NRS 0–10, mean (SD)	1808 / 123	2.5 (2.1)	1737/57	1.7 (1.9)	1645/83	0.8 (1.2)
PtGA, NRS 0–10, mean (SD)	1856 / 75	4.3 (2.6)	1697/97	3.6 (2.5)	1622/106	2.9 (2.4)
Pain (Patient), NRS 0–10, mean (SD)	1855 / 76	4.3 (2.6)	1404/390	3.9 (2.6)	1531/197	3.2 (2.5)
Fatigue (Patient), NRS 0–10, mean (SD)	1827 / 104	4.3 (2.9)	1399/395	4.3 (3.0)	NA	NA
Fatigue (Facit-f 0–10 scale), mean (SD)	NA	NA	NA	NA	125/1603	2.3
HAQ (0–3), mean (SD)	1841 / 90	0.8 (0.7)	NA	NA	NA	NA
FFbH, mean (SD)	NA	NA	1590/204	78.6 (22.0)	1261/467	78.0 (22.5)
DLQI, mean (SD)	1755 / 176	3.9 (5.2)	NA	NA	NA	NA
PsAID, mean (SD)	1722 / 209	3.2 (2.3)	NA	NA	551/1177	2.9 (2.2)
RAID, mean (SD)	NA	NA	1392/377	3.8 (2.4)	NA	NA
WHO-5, mean (SD)	1764 / 167	50.9 (24.2)	1381/413	52.4 (26.0)	NA	NA
WHO-5 moderate/severe, n (%)	1764 / 167	433 (24.5)	1381/413	357 (25.9)	NA	NA
PHQ-4, MW (SD)	NA	NA	NA	NA	1217/511	2.9 (2.9)
PHQ-4 moderate/severe, n (%)	NA	NA	NA	NA	1217/511	146 (14.6)

*DAPSA* disease activity in PsA, * DLQI* dermatology life quality index, * FFbH* funktionsfragebogen hannover,* HAQ* health assessment questionnaire,* NA* not available, * NDB* national data base, *PhGA* physician global disease activity, * PtGA* patient global disease activity, * PSAID* psoriatic arthritis impact of disease, * RABBIT-SpA* german disease register RABBIT-SpA, * RAID* rheumatoid arthritis impact of disease,* RHADAR* RheumaDatenRhePort, * SD* standard deviation,* WHO-5* WHO-5 well-being index

For better comparability, FACIT-f was transformed to 0–10 scale

### DMARD treatment

There were differences in the treatments that were prescribed in the three cohorts (Table 3). 76% of the RABBIT-SpA patients were on a b/tsDMARD treatment compared to 55% in NDB and 54% in RHADAR. csDMARD monotherapy was used in 13% in RABBIT-SpA, 29% in NDB, and 40% in RHADAR.

The distribution of the b/tsDMARDs was similar across the three cohorts. TNFi were most often used in all three cohorts; followed by IL-17i, IL-23i, IL-12/23i, and JAKi (Table 3).

**Table 3:** DMARD treatment

	*n* total/*n* missing	RABBIT-SpA	*n* total/*n* missing	NDB	*n* total/*n* missing	RHADAR
*n*		1931		1794		1728
b/tsDMARD, n (%)	1931/0	1459 (75.5)	1786/8	977 (54.7)	1021/707	614 (60.1)
TNFi, n (%)		586 (40.1)		470 (26.3)		310 (30.4)
IL-17i, n (%)		523 (35.8)		276 (15.5)		175 (17.1)
IL-23i, n (%)		134 (9.2)		63 (3.6)		42 (4.1)
IL-12/23i, n (%)		50 (3.4)		49 (2.8)		15 (1.5)
T-Cell Antagonist, n (%)		6 (0.4)		8 (0.5)		1 (0.1)
PDE-4i, n (%)		36 (2.5)		50 (2.8)		17 (1.7)
JAKi, n (%)		124 (8.5)		62 (3.5)		49 (4.8)
csDMARD mono, n (%)	1931/0	247 (12.8)	1786/8	524 (29.3)	1021/707	407 (39.9)

* b/tsDMARD* biologic/targeted synthetic disease modifying antirheumatic drug, * csDMARD* conventional synthetic DMARD, *TNFi* Tumor necrosis factor inhibitor,* IL* interleukin, * PDE-4i* Phosphodiesterase inhibitor, *JAKi* Janus kinase inhibitor

### Discordance of physician global disease activity compared to patient global disease activity

In RABBIT-SpA, the mean PhGA was 2.5 and the mean PtGA was 4.3; in NDB, the mean PhGA was 1.7 compared to 3.6 (mean PtGA) and in RHADAR, the mean PhGA was 0.85 compared to 2.9 (Table 2). The mean discordance (PtGA-PhGA) was 1.8 in RABBIT-SPA, 1.9 in NDB, and 2 in RHADAR.

Discordance > = 3 was present in 34% (RABBIT-SpA), 35% (NDB), and 37% (RHADAR); discordance of 1–2 in 33% (RABBIT-SpA), 35% (NDB), and 37% (RHADAR). Identical assessments were present in 18% (RABBIT-SpA), 22% (NDB), and 23% (RHADAR). In 15% (RABBIT-SpA), 8% (NDB), and 3% (RHADAR) the physicians rated higher values than the patients.

PtGA and PhGA are shown in Fig. 1. On the x-axis, PtGA values (0–10) are shown, and on the y-axis PhGA values. The boxplots show the distribution of PhGA values per PtGA value. For example, in the RABBIT-SpA cohort (Fig. 1a), in those 233 patients who reported a PtGA of 2, the median of PhGA was 1 with an interquartile range (IQR) of 0–2. The pink line shows the discordance between both medians, in this case, one. In those 166 patients with a PtGA of 8 the median PhGA was 4, the PhGA IQR ranged from 2 to 5 and the discordance was 4.

In all three cohorts there are similar features of the graph: for all PtGA values (0–10), the median PhGA is equal or lower and the discordance (pink line) between patient and physician values is increasing with higher PtGA values.

In the RABBIT-SpA cohort, the discordance was slightly smaller than in the NDB and in the RHADAR cohort. The largest discordance was found in RHADAR - this was also the cohort with the lowest PhGA values.

### Distribution of discordance between PtGA and PhGA

Figure 2 shows the magnitude of discordance between PtGA and PhGA per value of PtGA and the percentage of patients with the respective value. For example, in the RABBIT-SpA cohort, for those patients who reported a PtGA of 3, 19% had a discordance of > = 3 points, 43% had a discordance of 0–3 points, 21% of the physicians also rated 3, and 17% had higher values in their PhGA than 3.

It is evident that as the PtGA values increase, the discordance also increases. At PtGA 4, a discordance of > = 3 was present in 33% of the RABBIT-SpA patients, 49% of the NDB patients, and 71% of the RHADAR patients. This percentage increased progressively with higher PtGA values across all three cohorts. NDB exhibited higher and RHADAR even higher percentages of discordance > = 3 than RABBIT-SpA. In RHADAR, discordance was observed in the vast majority of patients with PtGA > = 4.

### Association between depressive symptoms and discordance between PtGA and PhGA

The association between the categories of depressive symptoms and the discordance is shown in Fig. 3. On the x-axis, the categories of depressive symptoms are shown. The light brown dot is the mean PhGA and the dark brown dot the mean PtGA; the red line shows the discordance between PhGA and PtGA. In those patients with severe depressive symptoms, the discordance between PtGA and PhGA was larger than in those with no depressive symptoms. The same trend was observed in all three cohorts.

To further analyse the relationship between PtGA and PhGA discordance and depressive symptoms, a regression model was applied. Age, sex and depressive symptoms, measured by WHO-5 in RABBIT-SpA and NDB and PHQ-4 in RHADAR, were included into the model in each cohort. Depressive symptoms were associated with discordance also if age and sex were accounted for in all cohorts (supplementary Table 1).

## Discussion

In this analysis of three independent cohorts of PsA patients, we found a clinically meaningful discordance between physician and patient assessed disease activity in more than a third of the patients. In all three cohorts, the discordance between physicians and patients assessment of global disease activity was associated with the presence of depressive symptoms.

The extent of the mean discordance of global disease activity found in our three cohorts is similar to previous reports [5]. Also the frequency is similar to previous studies [5–8]. Our results show that the frequency of discordance is rising sharply with increasing patients perceived disease activity. In cases where PtGA is > = 4, more than 30% of rheumatologists still rate the disease activity as low, and in PtGA > = 5, this number goes to more than 50%. These findings underscore the critical importance of acknowledging differences in perception when making shared treatment decisions.

The patients’ perception of disease activity seems different than that of the health care professionals (HCPs). While HCPs are trained to focus on objective inflammation markers, patients’ perceptions are influenced by a broader spectrum of factors like comorbidities (especially fibromyalgia), mental health, and cultural factors [13, 14]. Pain, the main PsA symptom, has different pathomechanisms with chronic inflammatory processes but also central sensitization playing an important role [14, 15]. Distinguishing between pain driven by active inflammation and pain stemming from other causes is essential for effective treatment choices [14].

Mental health disorders, particularly depression and anxiety, are widespread among PsA patients. In a previous analysis of the RABBIT-SpA cohort we found that 21% have moderate and 8% have severe depressive symptoms [16], while an analysis of the RHADAR cohort found a prevalence around 15% [17]. In this analysis we describe the association between the presence of depressive symptoms and discordance between PtGA and PhGA. In an earlier analysis of the RABBIT-SpA cohort, we showed similar findings in axSpA patients [16]. Similar results have also been described in a cohort of RA patients [18], in a cohort of patients admitted to hospital due to non-rheumatic diseases [19], and in psoriasis [20].

Discordance has also been described for other outcome parameter than global disease activity. For example, a large discordance between patients’ self-reported joint counts and rheumatologists clinical examination of the joints in PsA patients was found [21]. In an analysis of survey data from the US, misalignment between patient- and physician-reported satisfactions with PsA control was described and was associated with increased disease activity and disability [22].

Understanding the discordance between the PhGA and PtGA is crucial, particularly since the GRAPPA-OMERACT working group for standardizing outcome measure considers the shortened versions of the GRACE (3 visual analogue scale (VAS)/4VAS) in the process of finding a standard composite outcome measure for PsA [23]. The 3VAS consists of PhGA, PtGA and PtGA skin and the 4VAS includes PhGA, patient pain, PtGA joint and PtGA skin [24]. Both measures, 3VAS and 4VAS, demonstrated strong correlations and effective discrimination when compared to established composite measures [25]. However, it is unknown, to what extent the discordance between patients’ and physicians’ assessment influences these composite outcome scores. It is likely, that the discordance in the PhGA and PtGA as demonstrated by our study, can lead to misestimating disease activity, potentially distorting the overall picture, which is relevant for daily clinical practice as well as for observational studies and clinical trials.

One strength of our analysis is that it incorporates data from three independent cohorts of PsA patients, all which yielded consistent results. Nonetheless, some differences among the cohorts merit consideration. For example, the disease register RABBIT-SPA specifically includes patients requiring a new treatment after the failure of a first DMARD treatment, which explains higher disease activity and increased proportion of patients receiving b/tsDMARDs compared to those in the NDB and RHADAR cohorts. Additionally, the larger representation of university and highly specialized tertiary rheumatology centers in the RABBIT-SpA and NDB cohorts, relative to the RHADAR rheumatology network, further accounts for some of the observed differences.

This study has several limitations. The above described differences between the cohorts do not allow to analyse some of the potential confounder like comorbidities. Furthermore, we do not have any characteristics about the treating rheumatologists. It might be possible that for example age or gender of the physician has an impact on the PhGA. Our analysis is cross-sectional, only allowing us to describe associations without drawing causal conclusions. Nonetheless, the observed association between discordant assessments and mental health remains highly relevant for daily clinical practice. Such discordance can impact shared decision-making in chronic conditions, which depends on a mutual understanding of disease activity between patients and physicians. Misalignment in perceptions of disease activity and treatment outcomes may decrease patient satisfaction with therapeutic decisions, ultimately reducing adherence and treatment response.

Future research should aim to elucidate the underlying factors contributing to these discordances, including the roles of psychosocial factors, comorbidities, and varying manifestations of disease activity. Longitudinal studies are needed to determine how these differences in assessment impact treatment adherence, patient satisfaction, and long-term outcomes. Additionally, research into refining and standardizing assessment tools, as well as developing strategies to harmonize patient and physician evaluations could further improve clinical management [26].

## Conclusion

The frequency of clinically relevant discordance in our three real world cohorts is notably high. Moreover, as patients report higher levels of disease activity, discordant judgments by HCPs become increasingly prevalent. Additionally, depressive symptoms are strongly associated with discordant assessments of global disease activity. These findings underscore the need for HCP to be particularly mindful of patient-reported disease activity and depressive symptoms when evaluating disease status, as such factors may lead to discrepancies in clinical judgements and impact PsA management decisions. A deeper understanding of the mental health burden in PsA is crucial—not just for improving patient-physician communication but also for ensuring more effective, personalized treatment strategies.

## Supplementary Information

Below is the link to the electronic supplementary material.

Supplementary Material 1

## Data Availability

Data sharing: RABBIT-SpA data, NDB data, and Rhadar data is not approved to be shared.
