# Supplementary material for: Discordance between patient and physician reported global disease activity in PsA is associated with mental health—a cross-sectional analysis
Source: Rheumatol Int. 2025 Aug 5;45(8):181. doi: 10.1007/s00296-025-05933-0 (PMC12325493; doi:10.1007/s00296-025-05933-0)
Supplement: Supplementary file 1 — Supplementary Material 1 [file 296_2025_5933_MOESM1_ESM.docx]

Supplementary table 1: Results of linear regression models. The dependent variable is the difference between PtGA and PhGA.

|  | **RABBIT-SpA** | | | **NDB** | | | **RHADAR** | | |
| --- | --- | --- | --- | --- | --- | --- | --- | --- | --- |
| *Predictors* | *Estimates* | *95% CI* | *p* | *Estimates* | *95% CI* | *p* | *Estimates* | *95% CI* | *p* |
| (Intercept) | 2.61 | 1.92 – 3.29 | **<0.001** | 2.68 | 2.14 – 3.23 | **<0.001** | **1.34** | **0.75** – 1.93 | **<0.001** |
| Age, per year | 0.01 | -0.00 – 0.01 | 0.230 | 0.01 | 0.00 – 0.02 | **0.006** | **0.00** | **-0.01** – 0.01 | **0.757** |
| Sex (reference=female) | 0.01 | -0.22 – 0.25 | 0.921 | 0.04 | -0.19 – 0.27 | 0.714 | -0.19 | -0.43 – 0.06 | 0.138 |
| WHO-5 | -0.02 | -0.03 – -0.02 | **<0.001** | -0.03 | -0.03 – -0.02 | **<0.001** | **NA** | | |
| PHQ-4 | **NA** | | | **NA** | | | **0.36** | **0.32** – 0.41 | **<0.001** |
| Observations | 1653 | | | 1278 | | | 1002 | | |
| R^2^ / R^2^ adjusted | 0.051 / 0.049 | | | 0.099 / 0.097 | | | 0.229 / 0.226 | | |

Abbreviations: NA: not available; NDB: national data base; PhGA: physician global disease activity; PHQ-4: Patient health questionnaire, 4 Questions version; PtGA: patient global disease activity; RABBIT-SpA: German disease register RABBIT-SpA; RHADAR: RheumaDatenRhePort; WHO-5: WHO-5 Well-Being Index.
